# Modeling the effects of drug resistant influenza virus in a pandemic

**DOI:** 10.1186/1743-422X-5-133

**Published:** 2008-10-30

**Authors:** Stefan O Brockmann, Markus Schwehm, Hans-Peter Duerr, Mark Witschi, Daniel Koch, Beatriz Vidondo, Martin Eichner

**Affiliations:** 1 Department of Epidemiology and Health Reporting, Baden-Württemberg State Health Office, District Government Stuttgart, Germany; 2 Department of Medical Biometry, University of Tübingen, Germany; 3 Swiss Federal Office for Public Health, Bern, Switzerland

## Abstract

Neuraminidase inhibitors (NI) play a major role in plans to mitigate future influenza pandemics. Modeling studies suggested that a pandemic may be contained at the source by early treatment and prophylaxis with antiviral drugs. Here, we examine the influence of NI resistant influenza strains on an influenza pandemic. We extend the freely available deterministic simulation program *InfluSim *to incorporate importations of resistant infections and the emergence of *de novo *resistance. The epidemic with the fully drug sensitive strain leads to a cumulative number of 19,500 outpatients and 258 hospitalizations, respectively, per 100,000 inhabitants. Development of *de novo *resistance alone increases the total number of outpatients by about 6% and hospitalizations by about 21%. If a resistant infection is introduced into the population after three weeks, the outcome dramatically deteriorates. Wide-spread use of NI treatment makes it highly likely that the resistant strain will spread if its fitness is high. This situation is further aggravated if a resistant virus is imported into a country in the early phase of an outbreak. As NI-resistant influenza infections with high fitness and pathogenicity have just been observed, the emergence of drug resistance in treated populations and the transmission of drug resistant strains is an important public health concern for seasonal and pandemic influenza.

## Findings

Neuraminidase inhibitors (NI) play an important role in plans to mitigate future influenza pandemics [1]. Modeling studies suggested that a pandemic may be contained at the source, if treatment and prophylaxis are applied in an early phase of the epidemic. Large amounts of NI (mainly oseltamivir) have been stockpiled in many countries to prepare for pandemic influenza, and many national preparedness plans rely on this. However, recently doubts have been raised whether this strategy is realistic. Timeliness of the intervention due to difficulties in early recognition and logistic challenges are some of the points considered. The development of NI resistance is of further concern.

Influenza viruses undergo continuous genetic changes by means of mutation and recombination, promoting the emergence of drug resistant strains. Viral resistance may develop by modifications in the amino acid composition of the neuraminidase or in the affinity of haemagglutinin to the receptors of the cell surface [reviewed in [2]]. Prior to the 2007/8 influenza season, NI resistant strains were found in patients after treatment with oseltamivir and in patients not exposed to oseltamivir. Resistance to NI occurred at a low level: less then 1% of immuno-competent patients were found to be infected with resistant virus [3]. The emergence of a resistant strain may not necessarily be dangerous, as the "fitness" of the resistant strain determines its transmissibility [4,5]. Most resistant strains lacked "fitness" and were unlikely to spread, but early surveillance data from the 2007/8 influenza season on the northern hemisphere suggest that an oseltamivir resistant influenza virus type A(H1N1) circulates in several European countries and in the US [6,7]. The proportion of resistant infections ranges between 4% and 67% (mean 20%, approximately 1.700 tested isolates) and have been reported from 15 of 25 European countries under surveillance [8].

To obtain a better understanding of the consequences associated with the widespread use of NI as first-line option against a novel pandemic influenza strain, we extend the freely available simulation program InfluSim to simulate the emergence and spread of NI resistant strains [9,10]. We examine how the numbers of outpatients and hospitalizations change if resistance emerges de novo and is imported into a population in the early phase of an outbreak. We compare scenarios with and without the presence of drug resistance, using a basic reproduction number *R*_0 _of 2.5 [11]. *R*_0 _is the expected number of secondary infections per case in a completely susceptible population without interventions (it is calculated as the maximum eigenvalue of the next generation matrix) [12,13]. The fitness of the resistant infection, i.e. its capability to spread from person to person, is assumed to be the same as that of the drug sensitive one. Concordant to historical data and most pandemic plans [see [13,14]], we assume that one third of all infected individuals remain asymptomatic, one third becomes moderately sick and one third becomes severely sick and seeks medical help. All cases who seek medical help ('outpatients') are offered antiviral treatment, and we assume that the NI stockpile is sufficiently large. General (unspecified) social distancing measures [15,16] are simulated by reducing the number of contacts within the population by 10%. Isolation additionally reduces the number of contacts of moderately sick cases by 10%, of severe cases who stay at home by 20%, and of hospitalized cases by 30%. On day zero, a single infection (which is either drug sensitive or drug resistant) is introduced into a fully susceptible population. On day 21, a second introduction follows (again drug sensitive or resistant). Drug resistance is assumed to develop additionally *de novo *during the course of the pandemic wave (we assume that 4.1% of children and teenagers and 0.32% of adults [cf. [17-19]] infected with the drug sensitive virus develop a resistant infection when taking antiviral drugs. Cases infected with the resistant virus do no longer respond to antiviral treatment. We report the incidence and total number of outpatients and hospitalizations during the course of the pandemic wave in a Swiss population of 100,000 inhabitants. The emergence and the initial spread of drug resistance are highly stochastic. Deterministic simulations as those presented here give average or mean courses of the resulting dynamics, but do not show the full stochastic range of results.

Without drug resistance, the simulated influenza epidemic causes 19,500 outpatients and 258 hospitalizations per 100,000 inhabitants. If only drug sensitive infections are imported, and drug resistance develops only *de novo*, the number of outpatients increases to 20,700 (106%) and the number of hospitalizations increases to 312 (121%; Table 1). If resistant infections do not only develop *de novo*, but are imported into the population 21 days after onset of the epidemic, the numbers rise to 22,700 (116%) outpatients and to 420 (163%) hospitalizations. If the resistant strain is imported before the drug sensitive one, numbers even rise to 25,100 (129%) outpatients and 601 (233%) hospitalizations, (Table 1). The latter values do not change if the resistant strain is imported a second time on day 21.

**Table 1:** Expected number of outpatients and hospitalizations in various scenarios with drug resistant infections

1^st ^infection imported on day 0	2^nd ^infection imported on day 21	Total number of outpatients	Total number of hospitalizations
sensitive strain	sensitive strain	20,700	313
sensitive strain	resistant strain	22,700	420
resistant strain	sensitive strain	25,100	601
resistant strain	resistant strain	25,100	601

**Figure 1:**
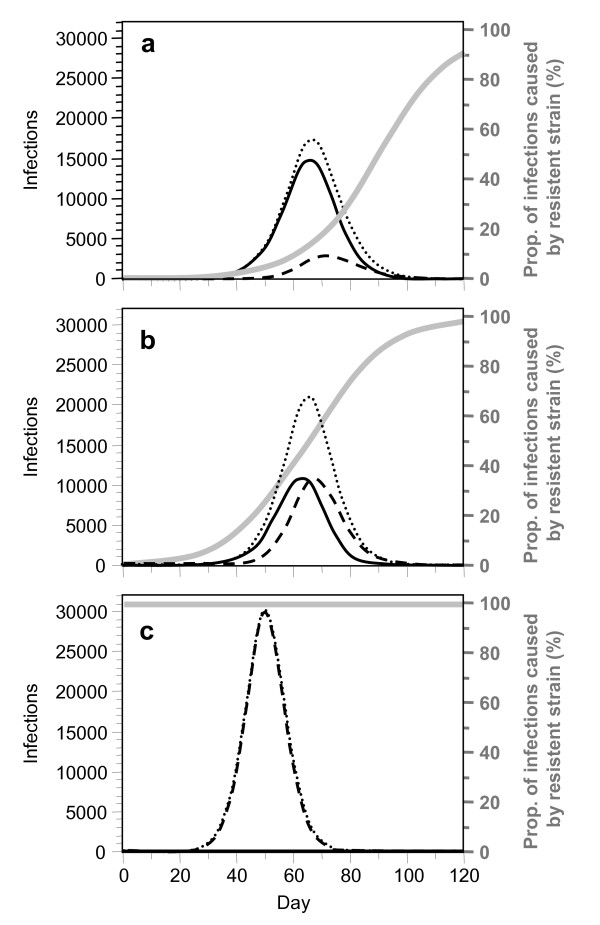
**Prevalence of infection with the drug sensitive virus (solid lines in black), the drug resistant one (dashed lines) and the sum of both (dotted lines).** All cases who seek medical help ('outpatients') receive antiviral treatment. The grey curves indicate the fractions of resistant infections among all infections. In all 3 graphs, resistance develops *de novo *in 4.1% of children and 0.32% of adults who receive treatment. (a) Drug-sensitive infections are imported on day 0 and 21; (b) Drug sensitive infection is imported on day 0, followed by a drug-resistant one on day 21; (c) Drug resistant infection is imported on day 0, followed by a drug-sensitive one on day 21. Further assumptions: (1) Swiss population of 100,000 individuals. (2) R_0 _= 2.5 for the drug sensitive and the drug resistant virus. Both strains are assumed to have the same transmissibility. (3) One third of all infected individuals become severely sick and seek medical help. Antiviral treatment reduces their contagiousness by 80% and their duration of sickness by 25% if they are infected with the drug sensitive virus. (4) General social distancing reduces the number of contacts by 10% for all individuals; isolation additionally prevents 10%, 20% and 30% of contacts of moderately sick cases, severely sick cases at home, and hospitalized cases, respectively. For references about assumptions and parameter values see text.

All patients who seek medical help ('outpatients') are offered antiviral treatment. The scenario without drug resistant infection leads to 19,500 outpatients and 258 hospitalizations, respectively. Population size 100,000; parameter values see Figure 1 and text.

**Figure 2:**
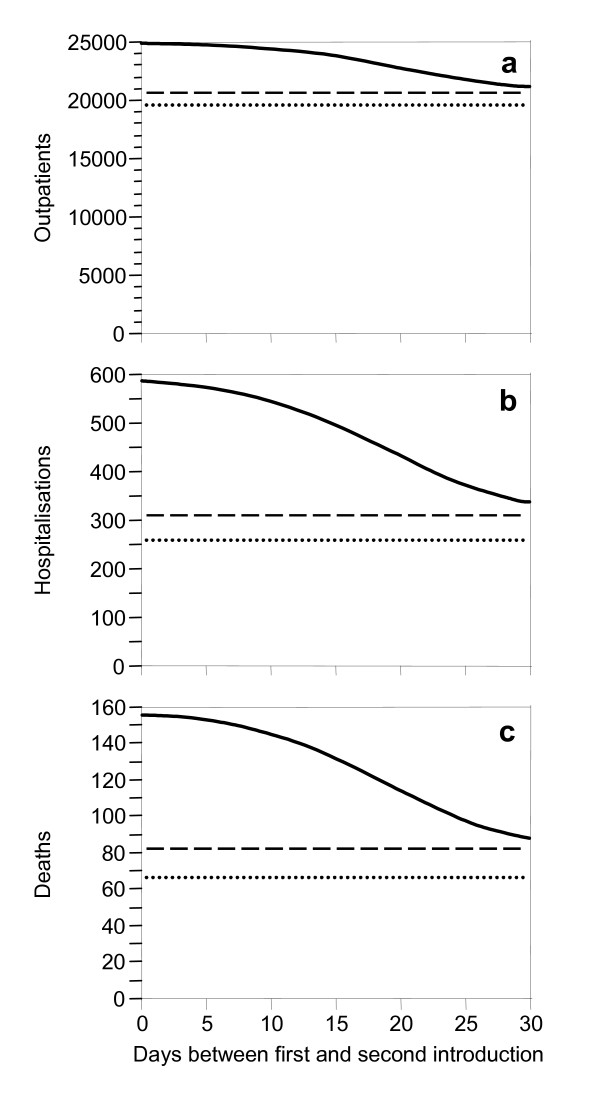
**The solid curves show the expected total numbers of (a) outpatients, (b) hospitalizations, and (c) deaths, respectively, during a pandemic wave in a population of 100,000 inhabitants where on day 0 a drug-sensitive infection is imported, followed by a drug-resistant one after the time delay given on the horizontal axis. **Without introduction of a resistant infection, 20,700 outpatients, 314 hospitalizations and 82 deaths are expected (dashed reference lines). If resistant infection is neither introduced *de novo *nor imported, 19,500 outpatients, 258 hospitalizations and 66 deaths are expected (dotted reference lines). Parameter values see Figure 1 and text.

If a resistant strain emerges only *de novo*, its prevalence may remain low, implying little epidemiological consequences (Figure 1a). Importation of resistance, however, increasingly replaces the drug sensitive strain because the latter is continuously eliminated by treatment. The dominance of the resistant strain depends on when its importation starts. E. g. if a drug resistant strain is imported 21 days after seeding the epidemic (with a sensitive strain), the prevalence curve for the resistant strain mimics in a delayed shape the prevalence of the sensitive strain (Figure 1b). If the time point for the importation of the resistant strain is shifted towards the initial phase of the epidemic, the resistant strain increasingly replaces the sensitive strain (Figure 1c). Early importation of resistant infection increases the number of treatment failures and thus, increases the overall number of infections emerging from the epidemic (Figure 1a–c). A sensitivity analysis which addresses the influence of the non-pharmaceutical interventions on these results is presented as an additional file (Additional file 1). Figure 2 illustrates the total numbers of (a) outpatients, (b) hospitalizations, and (c) deaths in dependence of a given time delay between the importation of the drug sensitive and the drug resistant infection (0–30 days).

Current mathematical models focus more on *de novo *drug resistance than on imported and spreading resistant infections [5,20,21]. Although *de novo *development of NI resistance may occur so late within a treated patient that the patient is unlikely to pass on the infection, wide-spread use of treatment makes it highly likely that resistant virus will circulate in the population if its relative fitness is high. We show in our simulations, that the development of *de novo *resistance on a low level and the subsequent spread of resistant virus results in a substantially increased number of hospitalizations, and subsequently in more ICU patients and deaths. Especially the shortcoming in the availability of intensive care beds has to be considered [22,23]. This situation is aggravated if an already resistant virus is imported into a population in the early phase of an epidemic. Up to now, only little attention has been paid to such scenarios. Observations in the early phase of the 2007/8 influenza season showed a marked increase of oseltamivir resistant influenza A virus (H1N1) in various European countries. The current oseltamivir resistant virus does not pose any risk to cause a pandemic as the H1N1 strain has been circulating in the population for many years without pandemic potential and leaving the population at least partially immune. The implications for avian influenza H5N1 remain uncertain. Oseltamivir resistance due to the same mutation has been reported in three patients with H5N1 infection who were treated with oseltamivir. As H5N1 viruses have not yet shown the ability to spread efficiently from person to person there seems currently no potential for a similar increase. However, the appearance of a spreading NI-resistant seasonal influenza strain is unexpected and of great concern. It highlights that even in the absence of widespread NI use for treatment or prophylaxis, oseltamivir resistant strains can emerge and spread in the population [6]. It also highlights the importance of our simulations for the elaboration of appropriate control and prevention strategies. We point out that the early introduction of a resistant influenza virus with pandemic potential may easily become an overwhelming public health problem. An increase of infections of 30% and a more than doubled total number of hospitalizations demonstrate this challenge. Non-pharmaceutical interventions considered by health decision makers and occupational medicine specialists in their pandemic preparedness plans may play a crucial role.

## List of abbreviations

NI: Neuraminidase inhibitors; *R*_0_: basic reproduction number.

## Competing interests

The authors declare that they have no competing interests.

## Authors' contributions

SOB and ME conceived the research question of the study, analyzed the simulation results and drafted the manuscript. ME and MS formulated and programmed the model in Java and delivered the simulation results. HPD participated in the design of the study, performed the statistical analysis, produced the figure and helped to draft the manuscript. DK, MW and BV participated in its design and coordination and helped to draft the manuscript. All authors read and approved the final manuscript.

## Supplementary Material

Additional File 1 **Sensitivity analysis on the influence of social distancing measures in the comparison of reintroduction of drug sensitive and drug resistant infection.** The data provided represent the sensitivity analysis on the influence of social distancing measures on the number of outpatients and hospitalizations. Click here for file
