# Supplementary material for: Modeling the effects of drug resistant influenza virus in a pandemic
Source: Virol J. 2008 Oct 30;5:133. doi: 10.1186/1743-422X-5-133 (PMC2590604; doi:10.1186/1743-422X-5-133)
Supplement: Additional File 1 — Sensitivity analysis on the influence of social distancing measures in the comparison of reintroduction of drug sensitive and drug resistant infection. The data provided represent the sensitivity analysis on the influence of social distancing measures on the number of outpatients and hospitalizations. [file 1743-422X-5-133-S1.doc]

# Sensitivity analysis on the influence of social distancing measures in the comparison of reintroduction of drug sensitive and drug resistant infection

Online supporting material to the paper ”Modeling the effects of drug resistant influenza virus in a pandemic“ by Stefan O. Brockmann, Markus Schwehm, Hans-Peter Duerr, Mark Witschi, Daniel Koch, Beatriz Vidondo, Martin Eichner

| general contact reduction by social distancing | Scenario A  Drug sensitive infection introduced on day 0 and on day 21 | | Scenario B Resistant infection introduced 21 days after drug sensitive infection | |
| --- | --- | --- | --- | --- |
|  | Number of outpatients | Number of hospitalizations | Number of outpatients  (increase) | Number of hospitalizations (increase) |
| 6 % | 21552 | 316 | 23045 (+6.9 %) | 399 (+26.3 %) |
| 8 % | 21132 | 314 | 22883 (+8.3 %) | 411 (+30.1 %) |
| 10 % | 20720 | 313 | 22728 (+9.7 %) | 423 (+35.1 %) |
| 12 % | 20326 | 314 | 22567 (+11.0 %) | 436 (+38.9 %) |
| 14% | 19960 | 316 | 22386 (+12.2 %) | 448 (+41.8 %) |
| 16 % | 19627 | 321 | 22170 (+13.0 %) | 458 (+42.7 %) |
| 18 % | 19334 | 327 | 21905 (+13.3 %) | 465 (+42.2 %) |
| 20 % | 19079 | 336 | 21578 (+13.1 %) | 470 (+39.9 %) |

Table S1: Simulation results of the increase in the number of outpatients and hospitalizations in a pandemic wave in 100,000 individuals if a resistant infection is introduced 21 days after the drug sensitive infection (Scenario B vs. Scenario A).

Interventions: (a) The general reduction of contacts is varied from 6 to 20%. (b) All cases are treated with antiviral drugs (they can develop resistance de novo as written in the text), (c) voluntary isolation reduces the contacts of moderately sick individuals by 10%, the contacts of severely sick patients who are taken care of at home by 20 % and the contacts with hospitalized cases by 30%.

| contact reduction of moderately sick / severely sick / hospitalized cases | Scenario A  Drug sensitive infection introduced on day 0 and on day 21 | | Scenario B Resistant infection introduced 21 days after drug sensitive infection | |
| --- | --- | --- | --- | --- |
|  | Number of outpatients | Number of hospitalizations | Number of outpatients  (increase) | Number of hospitalizations (increase) |
| 6/12/18 % | 21767 | 325 | 23338 (+7.2 %) | 412 (+26.8 %) |
| 8/16/24 % | 21257 | 319 | 23045 (+8.4 %) | 418 (+31.0 %) |
| 10/20/30 % | 20720 | 313 | 22728 (+9.7 %) | 423 (+35.1 %) |
| 12/24/36 % | 20154 | 307 | 22378 (+11.0 %) | 428 (+39.4 %) |
| 14/28/42% | 19557 | 300 | 21986 (+12.4 %) | 431 (+43.7 %) |
| 16/32/48 % | 18925 | 294 | 21538 (+13.8 %) | 432 (+46.9 %) |
| 18/36/54 % | 18255 | 286 | 21025 (+15.2 %) | 431 (+50.7 %) |
| 20/40/60 % | 17544 | 278 | 20433 (+16.5 %) | 427 (+53.6 %) |

Table S2: Simulation results of the increase in the number of outpatients and hospitalizations in a pandemic wave in 100,000 individuals if a resistant infection is introduced 21 days after the drug sensitive infection (Scenario B vs. Scenario A).

Interventions: (a) General reduction of contacts by 10%. (b) All cases are treated with antiviral drugs (they can develop resistance de novo as written in the text), (c) voluntary isolation reduces the contacts of moderately sick individuals, of severely sick patients who are taken care of at home, and of hospitalized cases by the fractions given in the left column.

Tables S1 and S2 show the detrimental effects of the introduction of the drug resistant pandemic strain of influenza three weeks after the introduction of a drug sensitive infection. The beneficial effect of social distancing measures or partial isolation of cases can be reduced by the introduction of resistant infection: the higher the gain which was obtained by contact reduction, the higher becomes the relative loss in case of a later reintroduction of a resistant infection.
